# Full Assignment of Ab-Initio Raman Spectra at Finite Temperatures Using Wannier Polarizabilities: Application to Cyclohexane Molecule in Gas Phase

**DOI:** 10.3390/mi12101212

**Published:** 2021-10-04

**Authors:** Pouya Partovi-Azar, Thomas D. Kühne

**Affiliations:** 1 Institute of Chemistry, Martin-Luther-University Halle-Wittenberg, Von-Danckelmann-Platz 4, D-06120 Halle (Saale), Germany; 2 Dynamics of Condensed Matter and Center for Sustainable Systems Design, Chair of Theoretical Chemistry, University of Paderborn, Warburger Str. 100, D-33098 Paderborn, Germany

**Keywords:** Raman spectrscopy, maximally localized Wannier functions, density functional theory, Car-Parrinello molecular dynamics

## Abstract

We demonstrate how to fully ascribe Raman peaks simulated using ab initio molecular dynamics to specific vibrations in the structure at finite temperatures by means of Wannier functions. Here, we adopt our newly introduced method for the simulation of the Raman spectra in which the total polarizability of the system is expressed as a sum over Wannier polarizabilities. The assignment is then based on the calculation of *partial* Raman activities arising from self- and/or cross-correlations between different types of Wannier functions in the system. Different types of Wannier functions can be distinguished based on their spatial spread. To demonstrate the predictive power of this approach, we applied it to the case of a cyclohexane molecule in the gas phase and were able to fully assign the simulated Raman peaks.

## 1. Introduction

ab initio simulations of vibrational spectra, where the electronic degrees of freedom are explicitly taken into account, often provide valuable information on the structure and dynamics of complex systems. Therefore, computer simulations nowadays represent an invaluable tool for the rationalization of, and as a complement to, experimental measurements. The key quantity in computing the vibrational spectrum of a system is its dipole moment. The dipole moment of a system can be unambiguously calculated using maximally localized Wannier functions (MLWFs) [1,2,3], which allow partitioning of the total electronic density into individual fragment contributions. In this, one can use the expectation value of the periodic position operator $\hat{r}$ in the Wannier representation in order to find the centers of the localized functions for arbitrary symmetries [4,5,6]. These Wannier functions are defined as

(1) $$w_{n}\left(r-R\right)=\frac{V}{{\left(2\pi \right)}^{3}}\int_{BZ}dk\;e^{-ik\cdot R}\sum_{m=1}^{J}U_{mn}^{\left(k\right)}\psi_{mk}\left(r\right),$$

where $\psi_{mk}\left(r\right)$ are Bloch functions, R is a Bravais lattice vector, and *V* the real-space primitive cell volume, while the integral is computed over the whole Brillouin zone [7]. The unitary J×J matrix $U_{mn}^{\left(k\right)}$ is periodic with respect to the wavevector k, while $\psi_{mk}\left(r\right)$ are the eigenstates of a system as obtained by an electronic structure method, such as density functional theory (DFT) [8]. To compute MLWFs, the total spread functional

(2) $$S=\sum_{n}S_{n}=\sum_{n}\left(\langle w_{n}\left|r^{2}\right|w_{n}\rangle -{\langle w_{n}\left|r\right|w_{n}\rangle }^{2}\right),$$

is minimized by appropriately chosen unitary rotations $U_{mn}^{\left(k\right)}$ [6].

By means of ab initio molecular dynamics (AIMD) simulations [9], one can obtain the isotropic Raman spectrum at finite temperature through the calculation of autocorrelation between polarizabilities of the system

(3) $$\sigma \left(\nu \right)\propto \nu \;\int_{0}^{\infty }dt\;e^{i2\pi \nu t}{\langle \bar{A}\left(0\right)\bar{A}\left(t\right)\rangle }_{\mathrm{cl}},$$

where ν is the frequency, $\bar{A}=\frac{1}{3}\mathrm{Tr}\left[\hat{A}\right]$ is the mean polarizability, and ${\langle \ldots \rangle }_{\mathrm{cl}}$ denotes the ensemble average of classical statistical mechanics [10,11]. Although Equation (3) includes finite temperature effects, the assignment of simulated peaks to Raman-active vibrations is not always straightforward. Recently, a computational technique was introduced to efficiently calculate Raman spectra [12,13]. In this, the polarizability is represented as a sum over Wannier polarizabilities, which are functions of Wannier orbital volumes and, consequently, their spatial spreads,

(4) $$\bar{A}=\frac{1}{3}\mathrm{Tr}\left[\hat{A}\right]=\frac{1}{3}\sum_{i}^{N_{\mathrm{WF}}}A_{i}=\frac{1}{3}\sum_{i}^{N_{\mathrm{WF}}}\beta S_{i}^{3},$$

with $S_{i}$ and $A_{i}$ being the spread of the *i*th Wannier function and its associated polarizability, respectively, whereas β is an empirical proportionality constant [12]. The present Wannier polarizability method has been employed in various systems, including gas-phase and liquid systems, and has been tested it for solids [12,13,14,15].

On the one hand, in computational methods, which are based on normal mode analysis [16,17], finite-temperature and anharmonic effects are omitted when simulating Raman spectra. On the other hand, however, the peak assignment is straightforward. Compared to these methods, the Wannier polarizability method has the advantage of including both effects. In addition, as will be demonstrated immediately, it also provides an attractive way to assign all spectroscopic peaks. In comparison to the other Raman simulation methods based on polarizability sampling (usually calculated through perturbative or finite-differences methods) [18,19,20,21,22] during AIMD simulations, the Wannier polarizability approach has a much lower computational cost. In fact, extending the second-generation Car-Parrinello method of Kühne et al. to allow for an on-the-fly localization of the electronic orbitals [23,24], MLWFs are dynamically generated during the AIMD simulation with negligible additional computational costs [12].

Here, we present a novel scheme to assign the ab initio Raman peaks to vibrations in the system at finite temperature using *partial* Raman spectra from individual, or a set of selected Wannier functions. As a proof of concept, we consider a cyclohexane molecule in the gas phase due to its simple covalent bonding that allows for a clear interpretation of its electronic charge distribution within a localized orbital representation. The resulting Wannier functions are localized along C–C and C–H bonds, which are easily distinguishable based on their spread. In spite of its simplicity, the cyclohexane molecule has a wealth of vibrations apart from those attributed to local bond stretching at higher frequencies, e.g., C–C–C bending, as well as wagging, rocking, scissoring, and twisting of CH${}_{2}$ groups. Our particular aim is to assess the Wannier polarizability-based peak assignment of these collective vibrations.

The remainder of the article in organized as follows. In Section 2, we outline the method we use for the calculation of the partial Raman spectrum. In Section 3, we present the fully assigned Raman spectrum of cyclohexane in the gas phase, and our observations and conclusions are presented in Section 4.

## 2. Methodology

The definition of the mean polarizability in Equation (4) allows for the calculation of partial Raman spectra by running the summation in Equation (4) through a selected set of Wannier polarizabilities $\left\{A_{1},A_{2},\ldots ,A_{M}\right\}$, namely

(5) $$\bar{A}=\frac{1}{3}\sum_{i=1}^{M\leq N_{\mathrm{WF}}}A_{i},$$

which for $M=N_{\mathrm{WF}}$ gives the total Raman spectrum. It also allows for decomposition of the time-correlation function in Equation (3) into self- and cross-correlations, i.e.,

(6) $$\langle \bar{A}\left(0\right)\bar{A}\left(t\right)\rangle =\langle \sum_{i}^{M}A_{i}\left(0\right)A_{i}\left(t\right)\rangle +\langle \sum_{i}^{M}\sum_{j(\neq i)}^{M}A_{i}\left(0\right)A_{j}\left(t\right)\rangle .$$

**Figure 1:**
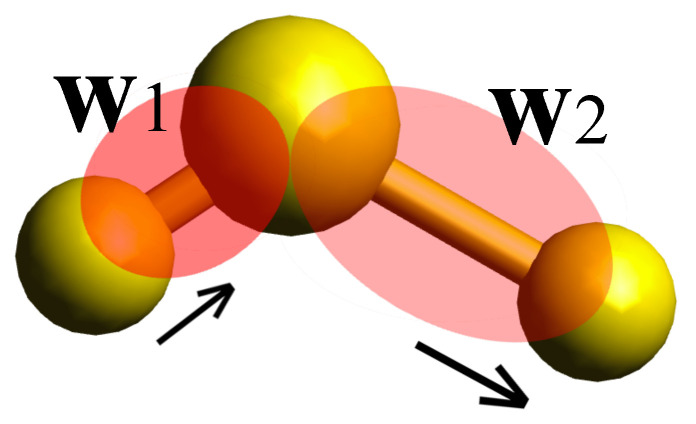
Schematic view of an asymmetric stretching mode of a molecule. Wannier functions $w_{1}$ and $w_{2}$, centered along two adjacent covalent bonds, are also shown.

As the Wannier functions are usually centered on bonds in covalent systems, the dynamics of the Wannier spreads give a clear picture of changes in the bond polarizability. Therefore, by calculating the autocorrelation of an individual Wannier polarizability via an AIMD simulation, one can obtain the contributions of bond stretching modes to the total spectrum. Similarly, the frequencies corresponding to bending, scissoring, wagging, and even those modes with higher participation ratios can be obtained by including two or more Wannier functions in Equation (5). For the calculation of individual stretching modes, only the self-correlation term in Equation (6) is required. However, in cases where two or more Wannier polarizabilities are included, the cross terms become important for the correct assignment of the peaks. Assuming that we are aiming for the assignment of symmetric and asymmetric stretching modes in a typical molecule, we need to include in Equation (5) the polarizabilities corresponding to two Wannier functions centered along two adjacent covalent bonds. This corresponds to set $\bar{A}=\frac{1}{3}\left(A_{1}+A_{2}\right)$, where $A_{1}$ and $A_{2}$ are the polarizabilities corresponding to Wannier functions $w_{1}$ and $w_{2}$ (see Figure 1).

When only one of the Wannier functions is considered, then $\bar{A}=\frac{1}{3}A_{1}$ (or $\bar{A}=\frac{1}{3}A_{2}$), the self-correlation term in Equation (6) would give rise to two distinct peaks in the calculated partial Raman spectrum due to symmetric and asymmetric vibrations of single bonds. However, when both Wannier functions are considered, the cross-term would eliminate the Fourier component corresponding to the asymmetric stretching mode, due to the fact that in such a vibration the spatial spread of Wannier functions anti-correlates with each other. Thus when one has the maximum spread, the other is fully contracted. This can be explicitly seen by assuming a very simple form for $A_{1}$ and $A_{2}$, for example $A_{1}=c_{1}\sin\left(2\pi \nu \right)+c_{2}\cos\left(2\pi \mu \right)$ and $A_{2}=c_{1}\sin\left(2\pi \nu \right)-c_{2}\cos\left(2\pi \mu \right)$, with ν and μ being the frequency of symmetric and asymmetric stretch, respectively. Anti-correlation would result in only one peak in the partial spectrum when $\bar{A}=\frac{1}{3}\left(A_{1}+A_{2}\right)$. Therefore, one can directly assign the peak that disappears to the asymmetric stretching mode. Through similar processes, all the vibrations can be attributed to Raman-active modes in the total spectrum, as will be demonstrated later for the case of a single cyclohexane molecule in chair conformation.

## 3. Computational Details

The minimum energy structure of a cyclohexane molecule in the chair conformation was obtained at the DFT level of theory using the mixed Gaussian and plane-wave code CP2K/Quickstep [25] in conjunction with a molecularly optimized double-zeta valence polarization Gaussian basis set [26], norm-conserving Goedecker-Teter-Hutter dual-space pseudopotentials [27], the Perdew-Burke-Ernzerhof exchange and correlation functional [28,29], as well as a semi-empirical correction for the long-range London dispersion interactions [30]. The electron density was represented on a regular plane-wave grid with a density cutoff of 280 Ry. To locate the nuclear ground state, the Broyden–Fletcher–Goldfarb–Shanno algorithm was employed till all nupositions and force were converged to 3.0 × 10${}^{-3}$ Bohr and 4.5 × 10${}^{-4}$ Hartree/Bohr, respectively [31,32,33,34]. The trajectory, necessary to compute finite-temperature Raman spectra, were obtained using the second-generation Car-Parrinello AIMD method of Kühne et al. [9,23], which had been modified so as to propagate in time not only the nuclei and electron density, but also the matrix $U_{mn}^{\left(k\right)}$ to automatically maintain the locality of MLWFs with essentially no additional computational cost [12,24]. The corresponding equations of motion [35], however, require the on-the-fly computation of nuclear and electronic gradients, as well as what we call “Wannier forces”. Using this scheme, together with a discretized time step of 0.5 fs, the system was equilibrated for 20 ps within the canonical ensemble, before the polarizabilities were sampled every 5 fs in the microcanonical ensemble for again 20 ps.

## 4. Results and Discussion

**Figure 2:**
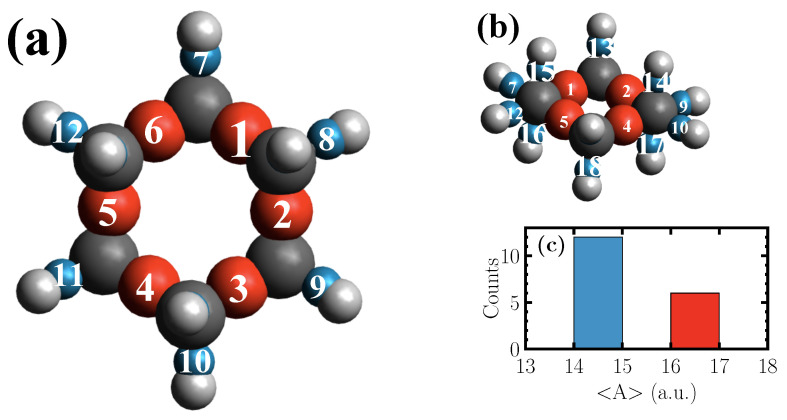
Atomic configuration of a cyclohexane molecule in its chair conformation shown by top (**a**) and side (**b**) views. The carbon and hydrogen atoms are shown as gray and white spheres, respectively. The centers of Wannier functions along C–C (red) and C–H (blue) bonds, are also shown. The radii of the spheres representing the Wannier functions are set according to their corresponding polarizabilities. Eventually, the polarizabilities averaged over all AIMD snapshots of the Wannier functions associated with C–C and C–H bonds are displayed in the inset (**c**).

The centers of the computed Wannier functions of the geometry optimized cyclohexane molecule, which in its chair conformation has $D_{3d}$ point group symmetry, are shown in Figure 2.

The red spheres correspond to the Wannier functions centered along C–C bonds, while the blue ones represent those along C–H bonds. The radii of the spheres are set according to the polarizability, which is shown in Figure 2c for the red and the blue spheres, respectively. As demonstrated, in the case of the molecule at hand, the Kohn-Sham eigenstates give rise to two types of Wannier functions which are distinguishable based on their polarizabilities. The frequencies of the fundamental vibrations of the simulated and experimental [36,37,38] Raman spectrum of a cyclohexane molecule in gas phase are listed in Table 1.

The fundamental frequency at 789 cm${}^{-1}$, ascribed to CH${}_{2}$ rocking vibrations, would only be seen for the crystalline phase [38]. It is also worth mentioning that the peak at 2923 cm${}^{-1}$ has been assigned to $e_{g}$, as well as to $a_{1g}$ vibrations [37]. The agreement of the simulated frequencies with the experimental results is generally good, with the exception of the high-frequency range. Even though the usage of a more accurate basis set would result in a better agreement in the frequency range higher than 2800 cm${}^{-1}$, even at the employed level of theory, the present method is rather useful for the accurate assignment of all Raman peaks.

**Figure 3:**
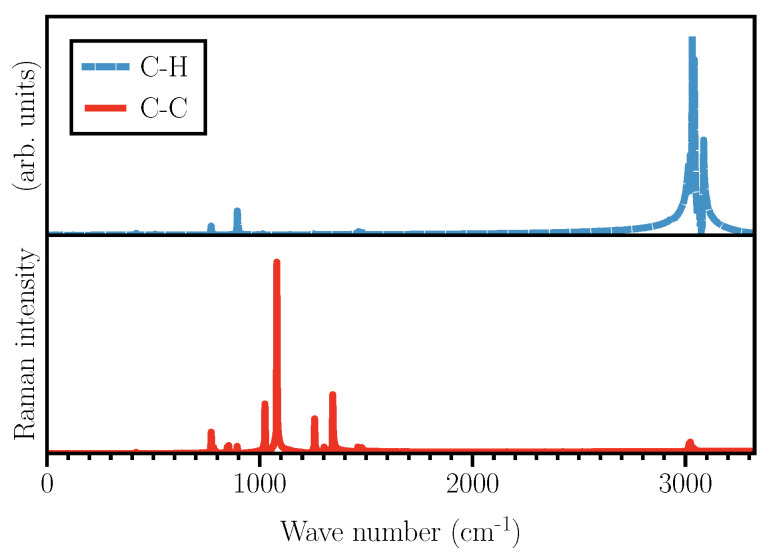
Simulated partial Raman spectra using Wannier functions along C–H (blue dashed) and C–C (red solid) bonds. Only the results obtained using just one Wannier function are shown for each type of bonds.

As shown in Figure 3, the two types of Wannier functions illustrated in Figure 2 also result in Raman activities within two different frequency ranges.

The activities shown in Figure 3 are obtained using typical Wannier functions along C–C and C–H bonds. Therefore, one needs to keep in mind that not all the activities shown are visible in the total Raman spectrum, as some of them might get suppressed when $M=N_{\mathrm{WF}}$ in Equation (5) due to the cancellation of Fourier components caused by a possible anti-correlation. Naively speaking, one might say that the activities around 3000 cm${}^{-1}$ should originate from C–H stretching modes, while the frequencies below 1500 cm${}^{-1}$ are due to C–C and ring vibrations. Although the vibrational spectrum of cyclohexane has been collected and studied for a long time, there are, even for such a simple molecule, some discrepancies regarding the assignment of the Raman peaks. For example, the peak at 1029 cm${}^{-1}$ has been ascribed to either CH${}_{2}$ rocking [36], or C–C stretching vibrations [38]. Here, by using Wannier polarizabilities, we can unambiguously assign this peak to C–C–C bending. In the following, we will assign all of the Raman modes listed in Table 1, one by one. Hereafter, C–X Wannier functions refer to those centered along C–X bonds.

The peak at 363 cm${}^{-1}$ is only present when single C–H Wannier functions are considered in Equation (5), but is not visible in the spectrum of single C–C Wannier functions. Thus, it should be a CH${}_{2}$ vibration. Nevertheless, the peak associated with single C–H Wannier functions undergoes a loss in intensity when both Wannier functions in a CH${}_{2}$ group are considered, which is a sign of partial anti-correlation arising from the cross term. Therefore, the mode cannot be a CH${}_{2}$ twisting vibration, since in that case complete anti-correlation would have been observed. It cannot be a wagging vibration either, since then two C–H bonds oscillate in-phase, hence the intensity should have been boosted instead of a partial intensity loss. As such, we attribute it to the rocking vibration of CH${}_{2}$ groups. This peak has been experimentally attributed to C–C–C deformation and C–C torsion [38], as well as to CH${}_{2}$ rocking vibrations [36].

The second peak at 420 cm${}^{-1}$ is absent in the spectra of single C–H Wannier functions within the plane of the cyclohexane ring shown in Figure 2a. However, an activity is observed in the spectra of single C–H Wannier functions above and below the ring plane (Figure 2b) at 420 cm${}^{-1}$. The activity is also present in the case of single C–C Wannier functions. Moreover, the peak gets boosted when two adjacent C–C Wannier functions are considered in Equation (5), which indicates an in-phase vibration. Nevertheless, the peak vanishes when three adjacent C–C Wannier functions are taken into account. This clearly indicates that the peak should correspond to a C–C–C bending, where two Wannier spreads change in-phase with each other, whereas the third Wannier spread is out-of-phase. At this frequency, the activity within the upper and lower C–H Wannier functions shows weak coupling of their spread to the motion of carbon atoms in the ring. This peak has experimentally also been attributed to C–C–C bending vibrations [36,38].

The next peak at 784 cm${}^{-1}$ exists in the spectra of single C–C Wannier functions. It is also visible when two, three, four, and five consecutive C–C Wannier functions are considered. In the case of five and six C–C Wannier functions, this peak gets considerably boosted, which points to the presence of in-phase collective vibrations of the carbon atoms in the ring. Therefore, it can be ascribed to the breathing mode of the ring. Experimentally, this peak has been attributed to C–C–C deformations [38], as well as to ring breathing [36].

The peak at 1024 cm${}^{-1}$ should arise from the C–C–C bending, since although it is visible in the spectra of all single C–C Wannier spreads, it vanishes when three consecutive C–C Wannier functions are included. It is also weakly present in the spectra of single C–H Wannier spreads within the ring plane, which arise from coupling between the C–C–C bending of the ring. It has previously been assigned to the CH${}_{2}$ rocking mode [36], and also to C–C stretching vibrations [38].

The following peak at 1140 cm${}^{-1}$ is present in the spectra of single C–C Wannier spreads. It is also visible in the ones of C–H Wannier functions above and below the plane of the ring, but only weakly present in the spectra of C–H Wannier functions in the ring plane. Therefore, it cannot be a wagging or a twisting vibration of the CH${}_{2}$ group. Moreover, it is present when more than one C–C Wannier function is included, so it is neither a stretching, nor a bending vibration of the ring. Hence, we assign this mode to ring deformations. Further information can be obtained by considering two C–H Wannier functions across the ring, one above the ring plane and the other below. The peak also appears in this case, which means that the C–H Wannier functions above the ring plane not only vibrate in an in-phase fashion with each other, they also oscillate in-phase with the C–H Wannier functions below. This immediately suggests a vibration of all C–H Wannier functions above and below the ring plane that move towards the center of the ring synchronously, which is likely accompanied by the movement of carbon atoms to form a flatter ring structure. This peak has been experimentally assigned to ring [38], and CH${}_{2}$ rocking vibrations [36].

The peak at 1258 cm${}^{-1}$ exists in the spectra of all C–H Wannier functions. Yet, the intensity of this peak is very low when single C–C Wannier functions are taken into account. Its intensity becomes even lower when two or three neighboring C–C Wannier functions are included. Therefore, it should arise from a CH${}_{2}$ group vibration. Moreover, the peak vanishes when two C–H Wannier functions belonging to single CH${}_{2}$ groups are considered, which indicates an out-of-phase oscillation of the corresponding C–H Wannier spreads. Consequently, we ascribe this peak to CH${}_{2}$ twisting vibration. Experimentally, this peak has indeed been ascribed to a CH${}_{2}$ twisting vibration [38], but it has also been assigned to CH${}_{2}$ wagging [36].

At 1344 cm${}^{-1}$, the vibration behaves quite similarly to the one at 1258 cm${}^{-1}$. It is present in single C–H Wannier functions above, below, and in the ring plane. Additionally, it is visible in single C–C Wannier functions. As in the case of the peak at 1258 cm${}^{-1}$, its intensity becomes lower as two and three neighboring C–C Wannier functions are included. However, the peak is also present when two C–H Wannier functions belonging to single CH${}_{2}$ groups are considered. Therefore, we assign it to CH${}_{2}$ wagging vibrations. This peak has been experimentally assigned to CH${}_{2}$ twisting [36], and wagging vibrations [38].

In the frequency range between 1400 to 1500 cm${}^{-1}$, there are two distinct peaks observed in the Raman spectrum, at 1460 and 1480 cm${}^{-1}$, respectively. Both peaks are present when single C–H Wannier functions in the ring plane are considered. The peak at 1480 cm${}^{-1}$ is also visible in the spectra of single C–H Wannier spreads above and below the ring plane. However, the peak at 1460 cm${}^{-1}$ can only be detected in the spectrum of one of the single C–H Wannier functions above and below the ring plane. Also, when two C–H Wannier functions in CH${}_{2}$ groups are considered, activity is profoundly seen around 1460 cm${}^{-1}$ in the case of two CH${}_{2}$ groups in front of each other across the ring. In the case of the other CH${}_{2}$ groups, the activity around this frequency is lower in intensity. Therefore, these two peaks should correspond to CH${}_{2}$ scissoring vibrations. Further investigations show that the two CH${}_{2}$ groups oscillate synchronously, while the other four CH${}_{2}$ groups are out-of-phase. It is worth mentioning that this rather complex vibration at 1460 cm${}^{-1}$ does not change the dipole moment of the molecule and is therefore IR-inactive.

The vibration at 1480 cm${}^{-1}$ is similar to the vibration at 1460 cm${}^{-1}$. However, in this case all CH${}_{2}$ scissoring vibrations oscillate synchronously. The reason is that the peak at 1480 cm${}^{-1}$ is not only present in the spectra of all single C–H Wannier functions, it is also visible in the spectra of two C–H Wannier functions in all CH${}_{2}$ groups.

The peaks above 3000 cm${}^{-1}$ are only present in the spectra of C–H Wannier functions. They are observed in the spectra of all single C–H Wannier functions at 3022, 3038, 3074, and 3089 cm${}^{-1}$, respectively. Hence, they should arise from the stretching mode of the C–H bonds. The first pair of peaks appear in the spectra of two C–H Wannier spreads in CH${}_{2}$ groups, while the latter pair vanish. This denotes that the first two correspond to symmetric (in-phase) stretching vibrations, whereas the latter two are due to asymmetric stretching. Furthermore, based on momentum conservation, we can assign the peak with lower frequency in each peak pair to out-of-phase stretching oscillations of CH${}_{2}$ groups across the ring. Yet, in order to maintain the position of the center of mass of the molecule in such oscillations, heavier carbon atoms also need to collectively oscillate, whereas in the in-phase stretching oscillations of CH${}_{2}$ groups, the molecular center of mass remains nearly fixed in space.

## 5. Conclusions

In this work, we have demonstrated the ability of our recently developed Wannier polarizability method to assign the Raman-active peaks to particular vibrations in the system. Here, the assignment has been based on the calculation of partial Raman activities arising from self- and/or cross-correlations between different Wannier functions in the system, which are differentiated based on their spatial spread. We have shown the applicability of this method in the case of a cyclohexane molecule in the gas phase. Yet, more complex condensed-phase systems can also be directly simulated using the present approach.

This work shows the advantages of the Wannier polarizability method not only in efficiently simulating the Raman spectra of general systems at finite temperatures [12,13], but also in unambiguously ascribing the computed Raman activities to specific vibrations. Therefore, the presented method is expected to be useful in a wide variety of applications.

## Figures and Tables

**Table 1:** Simulated Raman-active frequencies (in cm${}^{-1}$) of a cyclohexane molecule. In addition, experimental results from Ref. [38] are shown for comparison.

No.	Simulated	Exp. [38]	$D_{3d}$ Symm. [36,38]
1	363	383	$a_{1g}$
2	420	426	$e_{g}$
3	–	785	$e_{g}$
4	784	802	$a_{1g}$
5	1024	1027	$e_{g}$
6	1140	1157	$a_{1g}$
7	1258	1266	$e_{g}$
8	1344	1347	$e_{g}$
9	1460	1443	$e_{g}$
10	1480	1465	$a_{1g}$
11	3022	2852	$a_{1g}$
12	3038	2897	$e_{g}$
13	3074	2923	$a_{1g}$ ($e_{g}$ [37])
14	3089	2938	$a_{1g}$
